# A 10-Year Scientometrics Analysis of Brain Tumors Treated with Gamma Knife Radiosurgery: Visualization, Characteristics, and Scientific Trends

**DOI:** 10.1155/2022/7136868

**Published:** 2022-03-10

**Authors:** Walkiria Garcia-Fuentes, Leonardo Espinoza-Rodriguez, Arnaldo Munive-Degregori, Cesar Mauricio-Vilchez, Maria Eugenia Guerrero, Josmel Pacheco-Mendoza, Frank Mayta-Tovalino

**Affiliations:** ^1^School of Medicine, Faculty of Health Sciences, Universidad Científica del Sur, Lima, Peru; ^2^Department of Master's in Library and Information Science, Faculty of Arts and Human Sciences, Universidad Nacional Mayor de San Marcos, Lima, Peru; ^3^Postgraduate Department, Faculty of Dentistry, Universidad Nacional Federico Villarreal, Lima, Peru; ^4^Academic Department of Medical and Surgical Stomatology, Faculty of Dentistry, Universidad Nacional Mayor de San Marcos, Lima, Peru; ^5^Unidad de Investigación en Bibliometría, Universidad San Ignacio de Loyola, Lima, Peru

## Abstract

**Objective:**

To evaluate the scientometrics characteristics of the scientific production on the treatment of brain tumors with gamma knife radiosurgery in Scopus.

**Methods:**

The Scopus database (Elsevier) was used to collect all relevant studies for this bibliometric analysis. Data was obtained as a .csv file; it was downloaded from Scopus and was exported by SciVal to Microsoft Excel for a presentation using tables for more detailed analysis. The citations and the number of papers for the most productive institutions, authors, countries, and journals publishing scientific papers were analyzed on the use of gamma knife radiosurgery for brain tumors.

**Results:**

458 metadata were obtained from scientific publications, on which inclusion and exclusion criteria were applied, leaving 248 studies. The maximum peak of publications was 2018 with 31 publications, and the minimum peak was 2015 with 18. The most related subtopics were neurology, surgery, and oncology, and most of the retrieved papers had only institutional collaboration. As for more productive journals, *Journal of Neurosurgery* is first with 23 publications; within the countries with the most scientific publications, we have the United States, Japan, and China. With the United States being the country with the highest number of productions, University of Texas MD Anderson Cancer Center was the main university with the highest scientific production and Sheehan and Trifiletti and American authors dominate the list with the highest number of documents.

**Conclusion:**

Scientific production regarding gamma knife surgery and brain tumors has been increasing during the last 10 years, with a high incidence during 2018, and the highest number of published articles was in the first quartile. Likewise, the United States is the country with the most publications, authors, and universities on the subject. Greater efforts are still lacking from all countries in the world to reach the quantity and quality of production of the United States.

## 1. Introduction

Brain tumors are a common cause of mortality and morbidity [1, 2]. It is common that the choice of treatment for brain tumors is surgical resection, adding that by this, the definitive histological diagnosis can be established, but neurosurgery has been updating and improving its procedures over time, and currently the choice of treatment depends on the systemic condition of each patient [3]. In fact, gamma knife radiosurgery has become a new treatment modality since the first treatment was performed in Stockholm in 1968; since then, thousands of patients have undergone this procedure having an optimal result and follow-up, and long-term studies have been performed in order to perfect this treatment [4]. Indications for radiosurgery include brain tumors, arteriovenous malformations, brain metastases, malignant glioma, acoustic neuroma, and trigeminal neuralgia [3, 5].

Gamma knife radiosurgery, which can be an alternative or adjunctive treatment, has been shown to be effective, safe, and less invasive [5, 6]. This treatment does not require any incision; it is performed by the precise administration of large direct cytotoxic doses to targets within the brain; in this case, the tumor cells, of which their genetic material (DNA) is going to be damaged and therefore their ability to replicate will be affected, were able to decrease their size gradually without the need to invade the cranial vault, thus significantly decreasing intraoperative and postoperative risks [7]. Neurosurgeons considered it to be a single-session treatment, but constant research and therapeutic advances have considered reirradiation even of the same target, although it will require a greater effort for precision [8]. This treatment is still not performed in many countries since it can be considered a modern and updated treatment, but this article will evaluate the quality of the studies that have been carried out on this subject.

Scientometrics, which is responsible for the quantitative study of scientific and technical research activity, has bibliometrics as a branch, which focuses on scientific publications, estimating the publication patterns of the most cited articles based on an area of research [9, 10]. Pritchard established a definition in 1969, which is based on applying statistical and mathematical methods to studies [10, 11]. But it not only does involve the study of scientific publications and the citations presented but also has three main functions: description, evaluation of variables (number of scientific publications, citations of each author, institutions, etc.), and supervision or monitoring of research activity [10]. Therefore, this bibliometric analysis of articles based on brain tumors treated with gamma knife radiosurgery will allow us to evaluate and identify the scientific performance in this area, in which updates are increasing, to provide adequate objective information about this treatment through a critical analysis.

For that reason, the aim of this study was to perform a bibliometric analysis of the visualization, characteristics, and scientific trends in Scopus on gamma knife radiosurgery in patients with brain tumors, through a retrospective study analyzing publications in the last 10 years (2011-2020).

## 2. Methods

### 2.1. Database

**Figure 1:**
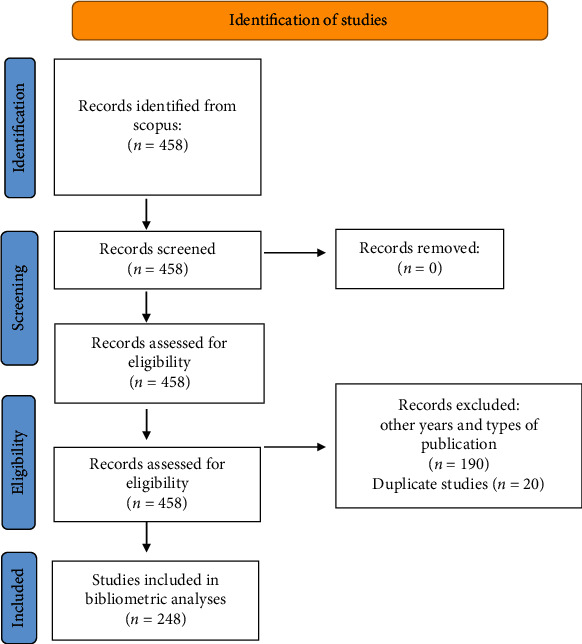
Flow diagram.

The Scopus database (Elsevier, USA) was used to collect all relevant studies for this bibliometric analysis. Scopus was chosen because it is a multidisciplinary database that collects studies at the international level, making it a large and reliable source of information. Likewise, Scopus offered the advantage of being able to work with SciVal, software used to calculate metrics and obtain more in-depth analysis for the bibliometric study, which was also made by Elsevier (Figure 1).

### 2.2. Search Strategy

First, a PICO strategy was created to obtain the keywords and their different variants were extracted from both Medical Subject Headings (MeSH) and Emtree from Embase, which together with the Boolean operators would serve to generate the search strategy that was run for the collection of information. The formula analyzed is attached at the end of the paper (Appendix S1). Scopus provided the option of filtering and limiting the results according to the type of study we wanted to consider. In this case, we only limited the information to articles and journals, excluding letters to the editor, notes, editorials, and conference proceedings; we also discarded all duplicate studies. Considering that SciVal can analyze studies from the last decade, we limited studies published only between 2011 and 2020. Studies outside the 2011-2020 cutoff dates were also excluded as before the date, the results did not represent a significant amount, and those from 2021 cannot yet be fully analyzed because at least 6 months are needed to update the Scopus database metrics.

### 2.3. Data Analysis

Scopus data was downloaded as a.csv file and then exported to Microsoft Excel for presentation using tables. The cutoff date was September 8, 2021. For more detailed analysis, to SciVal (Elsevier, USA) was exported the .csv file.

### 2.4. Bibliometric Indicators

The most productive authors, countries, journals, and institutions in relation to the use of gamma knife radiosurgery for brain tumors were analyzed. The main metrics used were (a) number of documents, (b) citations per publication, (c) impact (CiteScore), and (d) citations per countries, authors, and journals.

## 3. Results

### 3.1. Number and Impact of Publications

When placing the search strategy in Scopus, 458 metadata of scientific publications were found, on which inclusion and exclusion criteria were applied, leaving a total of 248 studies, of which there were in total 1446 authors, 3159 citations, and an average of 10.7 citations/document. The maximum peak of publications was 2018 with 31 publications, and the minimum peak was 2015 with 18 publications. Figure 1 shows the publications per year, where a trend of increasing publications can be seen especially between the years 2015 and 2020, but this does not follow a predictive pattern. According to the studies compiled by Scopus, 119 (47.9%) of these were in the top 25%, the quartile being considered an indicator that positions a journal within the total number of journals in its area according to the relative importance of each one. Within the top 76-100% are 11 studies, which, compared to the rest of the quartiles, has the lowest number of journals. The distribution of documents per year and the division by quartiles can be found in Table 1.

### 3.2. Thematic Areas of Gamma Knife Scientific Production

The papers collected had the highest incidence with the following subject areas: neurology (*n* = 138, 55.6%), surgery (*n* = 107, 43.1%), oncology (*n* = 93, 37.5%), and radiology, nuclear medicine, and imaging (*n* = 46, 18.5%). It should be considered that articles may have more than one subject area, so the sum of each area would give more than the total. The top 10 subject areas related to brain tumors and gamma knife radiosurgery are listed in Table 2.

### 3.3. Patterns of Collaboration of Gamma Knife Scientific Production

The majority of retrieved papers had only institutional collaboration (*n* = 108, 43.5%); then, came only national collaboration (*n* = 91, 36.7%) and international collaboration (*n* = 36, 14.5%). However, in terms of impact, although it was only institutional collaboration that had the most citations (*n* = 1255, 39.7%), international collaboration swept the citations per publication (1047 citations; 29.1 citations/document) surpassing even that of only institutional collaboration (1255 citations; 11.6 citations/document) and that of only national collaboration (91 citations; 978 citations/publication). The remaining collaborations, such as single-authored collaboration or no collaboration, represent 6.4% of the studies covering gamma knife radiosurgery in the treatment of brain tumors. All these analyses were performed on the data reflected in Table 3.

### 3.4. Top 10 Most Productive Scientific Journals

The 10 journals with the highest number of scientific publications on brain tumors and gamma knife surgery are shown in Table 4. Although there is not much difference between first and second place, the Journal of Neurosurgery is first with 23 publications (625 citations; 27.2 citations per publication) and in second place was World Neurosurgery with 20 publications (105 citations; 5.3 citations per publication); the third and fourth place goes to Journal of Neuro-Oncology with 13 publications (172 citations; 13.2 citations per publication) and Neurosurgery (121 citations; 11.0 citations per publication), respectively. However, the journals with the highest number of publications are not the same with the highest number of citations, much less with the number of citations per publication; under the latter concept appear Strahlentherapie and Onkologie with 40.3 citations per publication and Neuro-Oncology with 34.6 citations per publication. Table 4 shows the quartiles and how the journals have been classified according to them; it should be mentioned that a higher quartile does not necessarily indicate high quality of the publication.

### 3.5. Top 10 Universities

Table 5 shows the top 10 international universities with the highest number of publications on gamma knife radiosurgery. The University of Texas MD Anderson Cancer Center (United States), Harvard University (United States), and Stanford University (United States) were the 3 main universities with the highest scientific production (11, 10, and 10 publications, respectively). However, only Harvard University (United States) and University of Toronto (Canada) have had a bigger impact because they have the most citations with 378 and 251 citations, respectively.

### 3.6. Top 20 Most Productive Authors

The top 20 authors with the highest scientific production in gamma knife radiosurgery can be visualized in Table 6. Sheehan and Trifiletti, both from the United States, dominate the list with the highest number of papers (6 and 5, respectively), although there was not much difference between them. However, the most influential authors on gamma knife radiosurgery were Wen, Patrick Yung Chih; Hasegawa, Toshinori; and Kato, Takenori with 225, 194, and 194 citations, respectively (Table 6).

## 4. Discussion

Neurosurgery has been updated, improving its procedures over time, and currently the choice of treatment for brain tumors depends on the systemic condition of each patient, with one of the alternatives being gamma knife radiosurgery, being an updated treatment, which has proven to be effective, safe, and less invasive; for this reason, studies on this subject are currently increasing with the aim of obtaining improvements in the treatment, since with the passage of time, many patients have accepted to undergo this treatment [11, 12]; the goal of this study was to summarize all of the bibliometric properties of scientific articles published in the previous ten years.

In the present bibliometric study, the source of information was strictly from Scopus unlike other studies such as “The 100 Most Cited Papers About Brain Metastases”, which used Web of Science, SJR, Scopus, and Country rankings and InCites Journal Citation Reports [13]. However, this research has been more focused on filtering and ranking according to the selected parameters, excluding publications such as letters to the editor or conference proceedings that are not relevant to this study, collecting a total of 248 articles. A possible explanation for this is that, unlike the rest, this study focused only on analyzing quality and validated studies; the objective was not the quantity but rather the quality of each study included.

This study allowed us to perform a quantitative analysis of the activity in research, getting to know the number of scientific publications and number of citations presented, among other variables, about gamma knife radiosurgery and thus evaluate the scientific performance in this area. Although it was not a large number of articles analyzed, since it is a topic in which studies are just being conducted, these were sufficient to denote marked characteristics of the analysis; for example, the maximum peak of publications was in 2018 with 31 publications and the minimum peak was 2015 with 18 publications, and this increase as the years passed may be due to the great impact that gamma knife surgery began to take in the medical field during that year and the large increase in medical centers specialized in this branch of neurosurgery and research, thus expanding knowledge on the subject [14]. Other studies [13] showed variability in the number of publications per year, with a not so significant difference [15]. The increase in publications contributed to the growth of scientific literature about gamma knife radiosurgery, but further research on the topic is still needed. The number of collected articles is also lower compared to other bibliometric studies, because in this one, the search strategy was based on the association of gamma knife radiosurgery with brain tumors, and others are only based specifically on brain tumors, where a larger database is found [13, 14]. Some had as inclusion criteria articles with a longer publication period [12, 16], for example, with a publication date from 1899 to 2013 [17], and included animal studies.

High-income countries, such as the United States, China, and Japan, among others, had the highest productivity index, contributing to the growth of scientific literature. In the present study, the United States is the country that leads with 116 articles, the largest amount of scientific production on the subject, and this result coincides with several articles related to the subject [13, 17, 18], which could indicate that it is the country that is most interested in the advances and improvements of this medical treatment. It should also be noted that in addition to being the country with the highest scientific production, they also have the most citations, it would be logical to think that this is related to having the largest number of articles related to the topic and may be associated with the high reliability and quality of their studies, a common finding in several similar articles [13, 19].

Four universities in the United States occupy the top positions in terms of scientific production. The University of Texas ranks first with 11 published articles, followed by Harvard University and in third place Stanford University. Harvard University, despite being in the second place, has the highest number of citations, but the one who leads with the highest field-weighted citation impact is Ohio State University, which could indicate that they have a greater impact. This result differs slightly with other studies; in one study, the University of Wisconsin leads with 17 articles and the University of Texas is in the seventh place with 9 articles [13]; in another study [10, 19], the University of Pittsburgh leads with 46 articles and then the University of Virginia follows. This variable change is due to the fact that these studies are not only restricted to the topic of gamma knife radiosurgery associated with brain tumors, but some are associated with arteriovenous malformations in the brain [19] and others cover topics of brain metastasis [13], but we chose to make this comparison since they include gamma knife radiosurgery or brain tumors.

In this study, the level of international collaboration (14.3%) was not very frequent, but despite being a minimum percentage, it had many citations. In this study, the level of international collaboration was not very frequent (14.3%), although despite being a minimum percentage, it had many citations. On the other hand, the category of institutional collaboration led with a higher percentage (43%) and therefore had the highest number of citations (1255). The fact that international collaboration has many citations suggests that it is a very important point, which encourages the search for or creation of strategies to implement and increase the execution of this type of collaboration.

There are very few scientific publications on the subject up to the cutoff date, which does not allow it to be an extensive bibliometric study that is really synthesizing a large amount of data, but this does not affect the quality of the present study since the total population for that time has been analyzed anyway, and the fact that it is a small population does not mean that it is an insufficient study.

No publications from our region were found [20, 21], since this is a topic that has greater scope in first world countries, limiting the relevance of this research in our country. Most of the publications are from countries like the United States [13] and European countries; however, this does not mean that in the future, Latin America may make more progress in the implementation of gamma knife radiosurgery for brain tumors and that this study may have a greater impact.

There are also biases inherent to bibliometric studies such as very recent documents that may have limited citations, bias in the entities that group the publications by quartiles, and discordances between the names of the authors of the published journals. However, these errors would be the fault of the database rather than an error on our part as researchers since we tried to present the information objectively. Finally, this research was consistent with the methodology of other bibliometric articles [22–25].

## 5. Conclusions

It was concluded that the scientific production regarding gamma knife surgery and brain tumors has been increasing during the last 10 years, with a high incidence during 2018, and the highest number of articles was in the first quartile. Likewise, the United States was the country with the most publications, authors, and universities on the subject. Greater efforts are still lacking from all countries in the world to reach the quantity and quality of production of the United States.

## Figures and Tables

**Table 1:** All knife surgery publication types per journal quartile by CiteScore Percentile.

CiteScore quartile	Overall	2011	2012	2013	2014	2015	2016	2017	2018	2019	2020
Q1 (top 25%)	119	15	14	10	11	10	12	13	17	8	9
Q2 (top 26%-50%)	77	5	5	5	7	5	10	8	9	8	15
Q3 (top 51%-75%)	41	3	3	3	4	3	5	4	4	8	4
Q4 (top 76%-100%)	11	2	1	3	1	0	0	1	1	0	2
Total	248	25	23	21	23	18	27	26	31	24	30

**Table 2:** Top 10 subcategories by scholarly output of all publication types about knife surgery.

Subcategory	Scholarly output	Citations	Authors	Citations per publication	Field-weighted citation impact
Neurology (clinical)	138	1930	713	14	0.9
Surgery	107	1224	536	11.4	0.8
Oncology	93	1359	653	14.6	0.9
Radiology, nuclear medicine, and imaging	46	753	317	16.4	1.2
General medicine	11	31	47	2.8	0.2
Otorhinolaryngology	10	137	82	13.7	2
Hematology	8	61	75	7.6	0.7
Pediatrics, perinatology, and child health	7	50	34	7.1	0.5
Obstetrics and gynecology	3	15	16	5	0.4
Physiology (medical)	3	18	24	6	0.5

**Table 3:** All publication types in knife surgery by the amount of international, national, and institutional collaboration.

Metric	%	Scholarly output	Citations	Citations per publication	Field-weighted citation impact
International collaboration	14.3	36	1047	29.1	1.9
Only national collaboration	36.2	91	978	10.7	0.7
Only institutional collaboration	43.0	108	1255	11.6	0.7
Single authorship (no collaboration)	6.4	16	35	2.2	0.2

**Table 4:** Top 10 of all publication types of knife surgery by Scopus source.

Scopus source	Publications	Authors	Citations per publication	Source-Normalized Impact per Paper (SNIP)	CiteScore 2020	SCImago Journal Rank (SJR)
Journal of Neurosurgery	23	148	27.2	2	7.1	1.5
World Neurosurgery	20	92	5.3	1	2.9	0.7
Journal of Neuro-Oncology	13	75	13.2	1.2	6.4	1.2
Neurosurgery	11	54	11	1.7	6.6	1.4
International Journal of Radiation Oncology Biology Physics	10	89	25	1.8	9.1	2.1
Acta Neurochirurgica	10	53	7.8	1.2	3.2	0.7
Clinical Neurology and Neurosurgery	9	46	9.1	0.9	2.2	0.5
Neuro-Oncology	5	53	34.6	2.7	17.3	4
Radiation Oncology	5	32	26.8	1.2	4.8	1.0
Strahlentherapie und Onkologie	4	23	40.3	1	4.7	0.8
Journal of Cancer Research and Therapeutics	4	24	4.3	0.7	2.1	0.4

**Table 5:** Top 10 institutions (%) publishing about knife surgery.

Institution	Country	Scholarly output	Authors	Citations per publication	Field-weighted citation impact
University of Texas MD Anderson Cancer Center	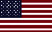	11	40	18.9	0.9
Harvard University	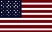	10	29	37.8	2.4
Stanford University	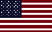	10	17	20.4	2
University of Virginia	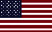	9	25	20.9	2.2
University of California at San Francisco	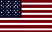	7	7	29.4	2.6
University of Toronto	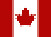	6	12	41.8	3.2
University of Pittsburgh	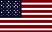	6	23	32.8	3.5
Capital Medical University	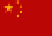	5	28	12.6	1
Ohio State University	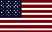	5	10	40	3.7
Thomas Jefferson University	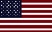	5	24	15.6	2.9

**Table 6:** Top 20 authors, by scholarly output in Scopus of all publication types about knife surgery.

Name	Scholarly output	Most recent publication	Citations	Citations per publication	Field-weighted citation impact	*h*-index
Sheehan, Jason P.	6	2020	109	18.2	1.1	58
Trifiletti, Daniel Michael	5	2020	80	16.0	1.2	19
Hasegawa, Toshinori	5	2020	194	38.8	2.0	23
Kato, Takenori	5	2020	194	38.8	2.0	8
Link, Michael J.	4	2017	104	26.0	1.3	47
Kano, Hideyuki	4	2019	150	37.5	2.5	41
Kida, Yoshihisa	4	2016	190	47.5	2.2	26
Shi, Wenyin	4	2020	20	5.0	0.5	25
Tuleasca, Constantin	3	2018	14	4.7	0.5	16
Carlson, Matthew Luke	3	2017	35	11.7	0.9	31
Levivier, Marc	3	2018	14	4.7	0.5	48
Lee, Chengchia	3	2016	87	29.0	1.5	25
Sahgal, Arjun A.	3	2020	38	12.7	0.9	51
Wen, Patrick Yung Chih	3	2016	225	75.0	2.2	96
Driscoll, Colin L.W.	3	2017	35	11.7	0.9	39
Lunsford, Lawrence Dade	3	2016	148	49.3	3.2	41
Li, Da	3	2019	52	17.3	1.5	13
Zhang, Liwei	3	2019	52	17.3	1.5	24
Şengöz, Meriç	3	2019	48	16.0	0.9	15
Zhang, Junting	3	2019	52	17.3	1.5	23

## Data Availability

The data used in the statistical analysis of this study will be available upon authorization of the corresponding author.

## Supplementary Materials

Supplementary Materials Appendix S1: search formula used.
