# Supplementary material for: A 10-Year Scientometrics Analysis of Brain Tumors Treated with Gamma Knife Radiosurgery: Visualization, Characteristics, and Scientific Trends
Source: J Cancer Epidemiol. 2022 Mar 10;2022:7136868. doi: 10.1155/2022/7136868 (PMC8930238; doi:10.1155/2022/7136868)
Supplement: Supplementary Materials — Appendix S1: search formula used. [file 7136868.f1.docx]

**Supplementary Materials**

Appendix S1: search formula used.

The SCOPUS search was performed by applying Boolean operators and keywords, with the following formula:

TITLE-ABS-KEY(“Gamma Knife Radiosurgery” OR “Radiosurgery Gamma Knife” OR “Gamma Knife Radiosurgeries” OR “Stereotactic Radiation” OR “Radiation Stereotactic” OR “Stereotactic Radiations” OR “Stereotactic Radiosurgery” OR “Radiosurgery Stereotactic” OR “Stereotactic Radiosurgeries” OR “Radiosurgery Linear Accelerator” OR “Linear Accelerator Radiosurgeries” OR “LINAC Radiosurgery” OR “LINAC Radiosurgeries” OR “Radiosurgery LINAC” OR “Linear Accelerator Radiosurgery” OR “Stereotactic Body Radiotherapy” OR “Radiotherapy Stereotactic Body” OR “Stereotactic Body Radiotherapies” OR “CyberKnife Radiosurgery” OR “CyberKnife Radiosurgeries” OR “Radiosurgery CyberKnife” OR “Stereotactic Radiation Therapy” OR “Radiation Therapy Stereotactic” OR “Stereotactic Radiation Therapies” OR “Therapy Stereotactic Radiation”) AND TITLE-ABS-KEY(“Brain Neoplasm” OR “Neoplasm Brain” OR “Neoplasms Brain” OR “Brain Tumors” OR “Brain Tumor” OR “Tumor Brain” OR “Benign Neoplasms Brain” OR “Benign Neoplasm Brain” OR “Brain Benign Neoplasm” OR “Brain Benign Neoplasms” OR “Neoplasms Brain, Benign” OR “Brain Neoplasms Benign” OR “Benign Brain Neoplasm” OR “Benign Brain Neoplasms” OR “Brain Neoplasm Benign” OR “Neoplasms Intracranial” OR “Intracranial Neoplasm” OR “Neoplasm Intracranial” OR “Intracranial Neoplasms” OR “Brain Tumor Primary” OR “Primary Brain Tumor” OR “Primary Brain Tumors” OR “Neoplasms Brain, Primary” OR “Brain Neoplasm Primary” OR “Primary Brain Neoplasms” OR “Brain Neoplasms Primary” OR “Primary Brain Neoplasm” OR “Brain Tumor Recurrent” OR “Brain Tumors Recurrent” OR “Recurrent Brain Tumor” OR “Recurrent Brain Tumors” OR “Malignant Primary Brain Tumors” OR “Primary Malignant Brain Tumors” OR “Malignant Primary Brain Neoplasms” OR “Primary Malignant Brain Neoplasms” OR “Brain Neoplasms Malignant Primary” OR “Brain Neoplasms Primary Malignant” OR “Brain Metastases” OR “Brain Metastase” OR “Brain Cancer” OR “Brain Cancers” OR “Cancer Brain” OR “Malignant Neoplasms Brain” OR “Brain Malignant Neoplasm” OR “Brain Malignant Neoplasms” OR “Malignant Neoplasm Brain” OR “Cancer of Brain” OR “Cancer of the Brain” OR “Neoplasms Brain Malignant” OR “Brain Neoplasms Malignant” OR “Brain Neoplasm Malignant” OR “Malignant Brain Neoplasm” OR “Malignant Brain Neoplasms” OR ‘‘Brain stem tumor’’)
